# Supplementary material for: Prognostic value of complementary biomarkers of neurodegeneration in a mixed memory clinic cohort
Source: PeerJ. 2020 Jul 9;8:e9498. doi: 10.7717/peerj.9498 (PMC7354835; doi:10.7717/peerj.9498)
Supplement: Supplemental Information 2 [file peerj-08-9498-s002.docx]

| **Lobe** | **Name** | **Extra info** |
| --- | --- | --- |
| Frontal Lobe | **Anterior Cingulate Gyrus** |  |
|  | **Anterior Insula** | separates frontal and temporal lobes (1&2) -> frontal |
|  | **Anterior Orbital Gyrus** |  |
|  | **Basal Forebrain** |  |
|  | **Central Operculum** |  |
|  | **Frontal Operculum** |  |
|  | **Frontal Pole** |  |
|  | **Gyrus Rectus** |  |
|  | **Lateral Orbital Gyrus** |  |
|  | **Medial Frontal Cortex** |  |
|  | **Medial Orbital Gyrus** |  |
|  | **Middle Cingulate Gyrus** |  |
|  | **Middle Frontal Gyrus** |  |
|  | **Opercular Part of the Inferior Frontal Gyrus** |  |
|  | **Orbital Part of the Inferior Frontal Gyrus** |  |
|  | **Posterior Orbital Gyrus** |  |
|  | **Precentral Gyrus Medial Segment** |  |
|  | **Precentral Gyrus** |  |
|  | **Subcallosal Area** |  |
|  | **Superior Frontal Gyrus** |  |
|  | **Superior Frontal Gyrus Medial Segment** |  |
|  | **Supplementary Motor Cortex** |  |
|  | **Triangular Part of the Inferior Frontal Gyrus** |  |
| Temporal Lobe | **Amygdala** | MTL |
|  | **Entorhinal Area** | MTL |
|  | **Hippocampus** | MTL |
|  | **Parahippocampal Gyrus** | MTL |
|  | **Fusiform Gyrus** | separates temporal & occipital lobes (2&4) -> temporal |
|  | **Inferior Temporal Gyrus** |  |
|  | **Middle Temporal Gyrus** |  |
|  | **Planum Polare** |  |
|  | **Planum Temporale** |  |
|  | **Superior Temporal Gyrus** |  |
|  | **Temporal Pole** |  |
|  | **Transverse Temporal Gyrus** |  |
| Parietal Lobe | **Angular Gyrus** |  |
|  | **Parietal Operculum** |  |
|  | **Postcentral Gyrus** |  |
|  | **Postcentral Gyrus Medial Segment** |  |
|  | **Posterior Cingulate Gyrus** | separates frontal & parietal lobes (1&3) -> parietal |
|  | **Posterior Insula** | separates parietal and temporal lobes (2&3) -> parietal |
|  | **Precuneus** |  |
|  | **Superior Parietal Lobule** |  |
|  | **Supramarginal Gyrus** |  |
| Occipital Lobe | **Calcarine Cortex** |  |
|  | **Cuneus** |  |
|  | **Inferior Occipital Gyrus** |  |
|  | **Lingual Gyrus** |  |
|  | **Middle Occipital Gyrus** |  |
|  | **Occipital Fusiform Gyrus** |  |
|  | **Occipital Pole** |  |
|  | **Superior Occipital Gyrus** |  |
| Ventricles | 3rd Ventricle |  |
|  | 4th Ventricle |  |
|  | CSF |  |
|  | **Inf Lat Vent** |  |
|  | **Lateral Ventricle** |  |
| Deep gray matter | **Accumbens Area** | GM |
|  | **Caudate** | GM |
|  | **Pallidum** | GM |
|  | **Putamen** | GM |
|  | **Thalamus Proper** | GM |
| Cerebellum | Cerebellar Vermal Lobules I-V | GM |
|  | Cerebellar Vermal Lobules VI-VII | GM |
|  | Cerebellar Vermal Lobules VIII-X | GM |
|  | **Cerebellum Exterior** | GM |
|  | **Cerebellum White Matter** | WM |
| Miscalleneous | Brain Stem | WM |
|  | **Cerebral Exterior** | GM |
|  | **Cerebral White Matter** | WM |
|  | Optic Chiasm | WM |
|  | **Ventral DC** | WM |
|  | **Vessel** | CSF |
