# Supplementary material for: Prognostic value of complementary biomarkers of neurodegeneration in a mixed memory clinic cohort
Source: PeerJ. 2020 Jul 9;8:e9498. doi: 10.7717/peerj.9498 (PMC7354835; doi:10.7717/peerj.9498)
Supplement: Supplemental Information 3 [file peerj-08-9498-s003.docx]

Sensitivity analyses

# Table 2

Abnormal markers full model -0.1/0.1

| Variable | Levels | Stable | Progression | Odds ratio (univariable) | Odds ratio (multivariable) |
| --- | --- | --- | --- | --- | --- |
| Age | Mean (SD) | 68.7 (8.9) | 71.1 (9.9) | 1.03 (0.98-1.09, p=0.272) | 1.02 (0.96-1.08, p=0.478) |
| Gender | Female | 24 (49.0) | 13 (48.1) | - | - |
|  | Male | 25 (51.0) | 14 (51.9) | 1.03 (0.40-2.65, p=0.945) | 1.09 (0.41-2.91, p=0.869) |
| Abnormal markers -0.1/0.1 | 0 | 18 (36.7) | 5 (18.5) | - | - |
|  | 1 | 16 (32.7) | 11 (40.7) | 2.47 (0.71-8.67, p=0.156) | 2.27 (0.63-8.17, p=0.209) |
|  | 2 | 12 (24.5) | 8 (29.6) | 2.40 (0.63-9.12, p=0.199) | 2.05 (0.50-8.36, p=0.315) |
|  | 3 | 3 (6.1) | 3 (11.1) | 3.60 (0.55-23.64, p=0.182) | 3.25 (0.48-21.96, p=0.226) |

Abnormal markers full model -0.5/0.5

| Variable | Levels | Stable | Progression | Odds ratio (univariable) | Odds ratio (multivariable) |
| --- | --- | --- | --- | --- | --- |
| Age | Mean (SD) | 68.7 (8.9) | 71.1 (9.9) | 1.03 (0.98-1.09, p=0.272) | 1.02 (0.96-1.08, p=0.529) |
| Gender | Female | 24 (49.0) | 13 (48.1) | - | - |
|  | Male | 25 (51.0) | 14 (51.9) | 1.03 (0.40-2.65, p=0.945) | 1.09 (0.41-2.90, p=0.869) |
| Abnormal markers 0.5/-0.5 sensitivity | 0 | 22 (44.9) | 7 (25.9) | - | - |
|  | 1 | 19 (38.8) | 16 (59.3) | 2.65 (0.90-7.79, p=0.077) | 2.35 (0.75-7.33, p=0.141) |
|  | 2 | 7 (14.3) | 4 (14.8) | 1.80 (0.40-8.00, p=0.442) | 1.62 (0.35-7.49, p=0.539) |
|  | 3 | 1 (2.0) | 0 (0.0) | 0.00 (0.00-Inf, p=0.992) | 0.00 (0.00-Inf, p=0.992) |

Abnormal markers simple model -0.1

| Variable | Levels | Stable | Progression | Odds ratio (univariable) | Odds ratio (multivariable) |
| --- | --- | --- | --- | --- | --- |
| Age | Mean (SD) | 68.6 (9.7) | 73.6 (9.2) | 1.06 (1.02-1.10, p=0.004) | 1.04 (1.00-1.09, p=0.068) |
| Gender | Female | 50 (50.5) | 27 (54.0) | - | - |
|  | Male | 49 (49.5) | 23 (46.0) | 0.87 (0.44-1.72, p=0.687) | 0.79 (0.37-1.66, p=0.529) |
| Abnormal MRI and FDG markers -0.1 sensitivity | 0 | 46 (46.5) | 10 (20.0) | - | - |
|  | 1 | 35 (35.4) | 24 (48.0) | 3.15 (1.34-7.44, p=0.009) | 2.57 (1.04-6.33, p=0.040) |
|  | 2 | 18 (18.2) | 16 (32.0) | 4.09 (1.57-10.68, p=0.004) | 3.12 (1.08-9.02, p=0.036) |

Abnormal markers simple model -0.5

| Variable | Levels | Stable | Progression | Odds ratio (univariable) | Odds ratio (multivariable) |
| --- | --- | --- | --- | --- | --- |
| Age | Mean (SD) | 68.6 (9.7) | 73.6 (9.2) | 1.06 (1.02-1.10, p=0.004) | 1.05 (1.00-1.10, p=0.030) |
| Gender | Female | 50 (50.5) | 27 (54.0) | - | - |
|  | Male | 49 (49.5) | 23 (46.0) | 0.87 (0.44-1.72, p=0.687) | 0.88 (0.42-1.84, p=0.739) |
| Abnormal MRI and FDG markers -0.5 sensitivity | 0 | 64 (64.6) | 20 (40.0) | - | - |
|  | 1 | 26 (26.3) | 27 (54.0) | 3.32 (1.59-6.94, p=0.001) | 2.51 (1.14-5.55, p=0.023) |
|  | 2 | 9 (9.1) | 3 (6.0) | 1.07 (0.26-4.32, p=0.928) | 0.70 (0.16-3.11, p=0.642) |

*Atrophy/hypometabolism patterns and progression - simple model (cut point z=-0.1)*

| Variable | Level | Stable | Progressed | OR (univariable) | OR (multivariable) |
| --- | --- | --- | --- | --- | --- |
| Age | Mean (SD) | 68.6 (9.7) | 73.6 (9.2) | 1.06 (1.02-1.10, p=0.004) | 1.04 (1.00-1.09, p=0.057) |
| Sex | Female | 50 (50.5) | 27 (54.0) | - | - |
|  | Male | 49 (49.5) | 23 (46.0) | 0.87 (0.44-1.72, p=0.687) | 1.44 (0.54-3.83, p=0.461) |
| +Atrophy, -Hypometabolism | 0-1 affected lobes | 60 (60.6) | 33 (66.0) | - | - |
|  | 2 or more affected lobes | 39 (39.4) | 17 (34.0) | 0.79 (0.39-1.61, p=0.521) | 1.10 (0.46-2.62, p=0.825) |
| -Atrophy, +Hypometabolism | 0-1 affected lobes | 69 (69.7) | 32 (64.0) | - | - |
|  | 2 or more affected lobes | 30 (30.3) | 18 (36.0) | 1.29 (0.63-2.66, p=0.483) | 1.16 (0.44-3.06, p=0.762) |
| +Atrophy, +Hypometabolism | 0-1 affected lobes | 72 (72.7) | 20 (40.0) | - | - |
|  | 2 or more affected lobes | 27 (27.3) | 30 (60.0) | 4.00 (1.95-8.20, p<0.001) | 3.75 (1.59-8.85, p=0.003) |

*Atrophy/hypometabolism patterns and progression in MCI/Dementia subcohort - simple model (cut point z=-0.1)*

| Variable | Level | Stable | Progressed | OR (univariable) | OR (multivariable) |
| --- | --- | --- | --- | --- | --- |
| Age | Mean (SD) | 71.6 (8.3) | 73.3 (8.9) | 1.02 (0.98-1.07, p=0.284) | 1.01 (0.96-1.06, p=0.694) |
| Sex | Female | 29 (44.6) | 26 (53.1) | - | - |
|  | Male | 36 (55.4) | 23 (46.9) | 0.71 (0.34-1.50, p=0.372) | 1.20 (0.42-3.40, p=0.737) |
| +Atrophy, -Hypometabolism | 0-1 affected lobes | 43 (66.2) | 33 (67.3) | - | - |
|  | 2 or more affected lobes | 22 (33.8) | 16 (32.7) | 0.95 (0.43-2.08, p=0.894) | 1.06 (0.42-2.70, p=0.904) |
| -Atrophy, +Hypometabolism | 0-1 affected lobes | 40 (61.5) | 31 (63.3) | - | - |
|  | 2 or more affected lobes | 25 (38.5) | 18 (36.7) | 0.93 (0.43-2.00, p=0.851) | 0.97 (0.37-2.56, p=0.951) |
| +Atrophy, +Hypometabolism | 0-1 affected lobes | 42 (64.6) | 20 (40.8) | - | - |
|  | 2 or more affected lobes | 23 (35.4) | 29 (59.2) | 2.65 (1.23-5.68, p=0.012) | 2.75 (1.09-6.92, p=0.032) |

*Isolated atrophy/hypometabolism patterns and progression(cut point z=-0.1)*

| Variable | Level | Stable | Progressed | OR (univariable) | OR (multivariable) |
| --- | --- | --- | --- | --- | --- |
| Age | Mean (SD) | 68.6 (9.7) | 73.6 (9.2) | 1.06 (1.02-1.10, p=0.004) | 1.04 (1.00-1.09, p=0.070) |
| Sex | Female | 50 (50.5) | 27 (54.0) | - | - |
|  | Male | 49 (49.5) | 23 (46.0) | 0.87 (0.44-1.72, p=0.687) | 1.30 (0.40-4.30, p=0.663) |
| +Atrophy, -Hypometabolism | 0-1 affected lobes | 60 (60.6) | 33 (66.0) | - | - |
|  | 2 or more affected lobes | 39 (39.4) | 17 (34.0) | 0.79 (0.39-1.61, p=0.521) | 2.09 (0.42-10.44, p=0.371) |
| -Atrophy, +Hypometabolism | 0-1 affected lobes | 69 (69.7) | 32 (64.0) | - | - |
|  | 2 or more affected lobes | 30 (30.3) | 18 (36.0) | 1.29 (0.63-2.66, p=0.483) | 0.51 (0.10-2.60, p=0.420) |
| +Atrophy, +Hypometabolism | 0-1 affected lobes | 72 (72.7) | 20 (40.0) | - | - |
|  | 2 or more affected lobes | 27 (27.3) | 30 (60.0) | 4.00 (1.95-8.20, p<0.001) | 3.05 (0.50-18.56, p=0.226) |
| Frontal isolated atrophy/hypometabolism | No abnormality | 32 (32.3) | 5 (10.0) | - | - |
|  | Any congruence and non-isolated incongruence | 19 (19.2) | 22 (44.0) | 7.41 (2.41-22.82, p<0.001) | 3.33 (0.49-22.81, p=0.220) |
|  | Isolated hypometabolism | 21 (21.2) | 13 (26.0) | 3.96 (1.23-12.75, p=0.021) | 1.90 (0.38-9.36, p=0.432) |
|  | Isolated atrophy | 27 (27.3) | 10 (20.0) | 2.37 (0.72-7.79, p=0.155) | 2.39 (0.36-16.03, p=0.369) |
| Temporal isolated atrophy/hypometabolism | No abnormality | 34 (34.3) | 7 (14.0) | - | - |
|  | Any congruence and non-isolated incongruence | 22 (22.2) | 25 (50.0) | 5.52 (2.04-14.93, p=0.001) | 4.22 (0.51-34.63, p=0.180) |
|  | Isolated hypometabolism | 19 (19.2) | 11 (22.0) | 2.81 (0.93-8.46, p=0.066) | 5.53 (0.73-41.65, p=0.097) |
|  | Isolated atrophy | 24 (24.2) | 7 (14.0) | 1.42 (0.44-4.57, p=0.560) | 2.16 (0.33-14.06, p=0.420) |
| Parietal isolated atrophy/hypometabolism | No abnormality | 32 (32.3) | 10 (20.0) | - | - |
|  | Any congruence and non-isolated incongruence | 25 (25.3) | 26 (52.0) | 3.33 (1.36-8.17, p=0.009) | 0.74 (0.14-3.88, p=0.725) |
|  | Isolated hypometabolism | 19 (19.2) | 9 (18.0) | 1.52 (0.52-4.39, p=0.444) | 1.16 (0.23-5.76, p=0.856) |
|  | Isolated atrophy | 23 (23.2) | 5 (10.0) | 0.70 (0.21-2.31, p=0.553) | 0.48 (0.08-2.97, p=0.431) |
| Occipital isolated atrophy/hypometabolism | No abnormality | 22 (22.2) | 13 (26.0) | - | - |
|  | Any congruence and non-isolated incongruence | 29 (29.3) | 19 (38.0) | 1.11 (0.45-2.72, p=0.822) | 0.12 (0.02-0.57, p=0.008) |
|  | Isolated hypometabolism | 18 (18.2) | 10 (20.0) | 0.94 (0.33-2.64, p=0.907) | 0.35 (0.08-1.62, p=0.179) |
|  | Isolated atrophy | 30 (30.3) | 8 (16.0) | 0.45 (0.16-1.27, p=0.133) | 0.11 (0.02-0.64, p=0.014) |

*Isolated atrophy/hypometabolism patterns and progression in MCI/dementia (cut point z=-0.1)*

| Variable | Level | Stable | Progressed | OR (univariable) | OR (multivariable) |
| --- | --- | --- | --- | --- | --- |
| Age | Mean (SD) | 71.6 (8.3) | 73.3 (8.9) | 1.02 (0.98-1.07, p=0.284) | 1.02 (0.96-1.08, p=0.508) |
| Sex | Female | 29 (44.6) | 26 (53.1) | - | - |
|  | Male | 36 (55.4) | 23 (46.9) | 0.71 (0.34-1.50, p=0.372) | 1.45 (0.38-5.52, p=0.587) |
| +Atrophy, -Hypometabolism | 0-1 affected lobes | 43 (66.2) | 33 (67.3) | - | - |
|  | 2 or more affected lobes | 22 (33.8) | 16 (32.7) | 0.95 (0.43-2.08, p=0.894) | 3.43 (0.53-22.35, p=0.198) |
| -Atrophy, +Hypometabolism | 0-1 affected lobes | 40 (61.5) | 31 (63.3) | - | - |
|  | 2 or more affected lobes | 25 (38.5) | 18 (36.7) | 0.93 (0.43-2.00, p=0.851) | 0.48 (0.09-2.60, p=0.397) |
| +Atrophy, +Hypometabolism | 0-1 affected lobes | 42 (64.6) | 20 (40.8) | - | - |
|  | 2 or more affected lobes | 23 (35.4) | 29 (59.2) | 2.65 (1.23-5.68, p=0.012) | 1.78 (0.25-12.51, p=0.565) |
| Frontal isolated atrophy/hypometabolism | No abnormality | 17 (26.2) | 5 (10.2) | - | - |
|  | Any congruence and non-isolated incongruence | 15 (23.1) | 22 (44.9) | 4.99 (1.51-16.45, p=0.008) | 7.34 (0.80-67.31, p=0.078) |
|  | Isolated hypometabolism | 17 (26.2) | 13 (26.5) | 2.60 (0.76-8.91, p=0.128) | 2.57 (0.51-13.05, p=0.254) |
|  | Isolated atrophy | 16 (24.6) | 9 (18.4) | 1.91 (0.53-6.94, p=0.324) | 2.96 (0.33-26.89, p=0.336) |
| Temporal isolated atrophy/hypometabolism | No abnormality | 18 (27.7) | 7 (14.3) | - | - |
|  | Any congruence and non-isolated incongruence | 20 (30.8) | 24 (49.0) | 3.09 (1.07-8.87, p=0.036) | 3.21 (0.28-37.26, p=0.352) |
|  | Isolated hypometabolism | 14 (21.5) | 11 (22.4) | 2.02 (0.62-6.56, p=0.242) | 5.93 (0.65-54.16, p=0.115) |
|  | Isolated atrophy | 13 (20.0) | 7 (14.3) | 1.38 (0.39-4.92, p=0.615) | 2.19 (0.22-21.99, p=0.505) |
| Parietal isolated atrophy/hypometabolism | No abnormality | 15 (23.1) | 10 (20.4) | - | - |
|  | Any congruence and non-isolated incongruence | 20 (30.8) | 26 (53.1) | 1.95 (0.72-5.25, p=0.186) | 0.61 (0.09-4.09, p=0.607) |
|  | Isolated hypometabolism | 17 (26.2) | 9 (18.4) | 0.79 (0.25-2.48, p=0.691) | 0.68 (0.13-3.62, p=0.650) |
|  | Isolated atrophy | 13 (20.0) | 4 (8.2) | 0.46 (0.12-1.83, p=0.271) | 0.37 (0.05-3.08, p=0.361) |
| Occipital isolated atrophy/hypometabolism | No abnormality | 12 (18.5) | 13 (26.5) | - | - |
|  | Any congruence and non-isolated incongruence | 23 (35.4) | 19 (38.8) | 0.76 (0.28-2.06, p=0.592) | 0.13 (0.02-0.74, p=0.021) |
|  | Isolated hypometabolism | 15 (23.1) | 10 (20.4) | 0.62 (0.20-1.89, p=0.396) | 0.34 (0.06-1.90, p=0.220) |
|  | Isolated atrophy | 15 (23.1) | 7 (14.3) | 0.43 (0.13-1.42, p=0.166) | 0.06 (0.01-0.54, p=0.011) |

Atrophy/hypometabolism patterns and progression - simple model (cut point z=-0.5)

| Variable | Level | Stable | Progressed | OR (univariable) | OR (multivariable) |
| --- | --- | --- | --- | --- | --- |
| Age | Mean (SD) | 68.6 (9.7) | 73.6 (9.2) | 1.06 (1.02-1.10, p=0.004) | 1.05 (1.01-1.10, p=0.013) |
| Sex | Female | 50 (50.5) | 27 (54.0) | - | - |
|  | Male | 49 (49.5) | 23 (46.0) | 0.87 (0.44-1.72, p=0.687) | 0.92 (0.37-2.27, p=0.858) |
| +Atrophy, -Hypometabolism | 0-1 affected lobes | 62 (62.6) | 35 (70.0) | - | - |
|  | 2 or more affected lobes | 37 (37.4) | 15 (30.0) | 0.72 (0.35-1.49, p=0.373) | 0.69 (0.29-1.64, p=0.399) |
| -Atrophy, +Hypometabolism | 0-1 affected lobes | 74 (74.7) | 33 (66.0) | - | - |
|  | 2 or more affected lobes | 25 (25.3) | 17 (34.0) | 1.52 (0.73-3.20, p=0.264) | 1.02 (0.42-2.45, p=0.968) |
| +Atrophy, +Hypometabolism | 0-1 affected lobes | 80 (80.8) | 33 (66.0) | - | - |
|  | 2 or more affected lobes | 19 (19.2) | 17 (34.0) | 2.17 (1.00-4.68, p=0.049) | 1.62 (0.66-3.99, p=0.292) |

Atrophy/hypometabolism patterns and progression in MCI/Dementia subcohort - simple model (cut point z=-0.5)

| Variable | Level | Stable | Progressed | OR (univariable) | OR (multivariable) |
| --- | --- | --- | --- | --- | --- |
| Age | Mean (SD) | 71.6 (8.3) | 73.3 (8.9) | 1.02 (0.98-1.07, p=0.284) | 1.02 (0.97-1.07, p=0.412) |
| Sex | Female | 29 (44.6) | 26 (53.1) | - | - |
|  | Male | 36 (55.4) | 23 (46.9) | 0.71 (0.34-1.50, p=0.372) | 0.56 (0.20-1.58, p=0.274) |
| +Atrophy, -Hypometabolism | 0-1 affected lobes | 39 (60.0) | 34 (69.4) | - | - |
|  | 2 or more affected lobes | 26 (40.0) | 15 (30.6) | 0.66 (0.30-1.45, p=0.302) | 0.46 (0.17-1.23, p=0.122) |
| -Atrophy, +Hypometabolism | 0-1 affected lobes | 43 (66.2) | 32 (65.3) | - | - |
|  | 2 or more affected lobes | 22 (33.8) | 17 (34.7) | 1.04 (0.48-2.27, p=0.925) | 0.85 (0.34-2.11, p=0.723) |
| +Atrophy, +Hypometabolism | 0-1 affected lobes | 48 (73.8) | 32 (65.3) | - | - |
|  | 2 or more affected lobes | 17 (26.2) | 17 (34.7) | 1.50 (0.67-3.36, p=0.325) | 1.09 (0.41-2.87, p=0.863) |

Isolated atrophy/hypometabolism patterns and progression (cut point z=-0.5)

| Variable | Level | Stable | Progressed | OR (univariable) | OR (multivariable) |
| --- | --- | --- | --- | --- | --- |
| Age | Mean (SD) | 68.6 (9.7) | 73.6 (9.2) | 1.06 (1.02-1.10, p=0.004) | 1.07 (1.02-1.12, p=0.010) |
| Sex | Female | 50 (50.5) | 27 (54.0) | - | - |
|  | Male | 49 (49.5) | 23 (46.0) | 0.87 (0.44-1.72, p=0.687) | 0.99 (0.33-2.96, p=0.989) |
| +Atrophy, -Hypometabolism | 0-1 affected lobes | 62 (62.6) | 35 (70.0) | - | - |
|  | 2 or more affected lobes | 37 (37.4) | 15 (30.0) | 0.72 (0.35-1.49, p=0.373) | 0.28 (0.05-1.67, p=0.163) |
| -Atrophy, +Hypometabolism | 0-1 affected lobes | 74 (74.7) | 33 (66.0) | - | - |
|  | 2 or more affected lobes | 25 (25.3) | 17 (34.0) | 1.52 (0.73-3.20, p=0.264) | 0.86 (0.13-5.64, p=0.873) |
| +Atrophy, +Hypometabolism | 0-1 affected lobes | 80 (80.8) | 33 (66.0) | - | - |
|  | 2 or more affected lobes | 19 (19.2) | 17 (34.0) | 2.17 (1.00-4.68, p=0.049) | 0.42 (0.05-3.83, p=0.443) |
| Frontal isolated atrophy/hypometabolism | No abnormality | 51 (51.5) | 19 (38.0) | - | - |
|  | Any congruence and non-isolated incongruence | 10 (10.1) | 13 (26.0) | 3.49 (1.31-9.28, p=0.012) | 1.72 (0.26-11.37, p=0.571) |
|  | Isolated hypometabolism | 15 (15.2) | 9 (18.0) | 1.61 (0.60-4.29, p=0.341) | 0.39 (0.07-2.18, p=0.282) |
|  | Isolated atrophy | 23 (23.2) | 9 (18.0) | 1.05 (0.41-2.67, p=0.918) | 0.76 (0.15-3.77, p=0.738) |
| Temporal isolated atrophy/hypometabolism | No abnormality | 52 (52.5) | 18 (36.0) | - | - |
|  | Any congruence and non-isolated incongruence | 14 (14.1) | 16 (32.0) | 3.30 (1.35-8.08, p=0.009) | 4.98 (0.63-39.45, p=0.129) |
|  | Isolated hypometabolism | 12 (12.1) | 10 (20.0) | 2.41 (0.89-6.52, p=0.084) | 2.35 (0.49-11.30, p=0.288) |
|  | Isolated atrophy | 21 (21.2) | 6 (12.0) | 0.83 (0.29-2.37, p=0.721) | 0.77 (0.15-3.85, p=0.751) |
| Parietal isolated atrophy/hypometabolism | No abnormality | 55 (55.6) | 14 (28.0) | - | - |
|  | Any congruence and non-isolated incongruence | 13 (13.1) | 15 (30.0) | 4.53 (1.76-11.68, p=0.002) | 15.67 (1.93-127.24, p=0.010) |
|  | Isolated hypometabolism | 12 (12.1) | 10 (20.0) | 3.27 (1.18-9.11, p=0.023) | 6.04 (0.94-38.97, p=0.059) |
|  | Isolated atrophy | 19 (19.2) | 11 (22.0) | 2.27 (0.88-5.86, p=0.089) | 14.11 (2.48-80.29, p=0.003) |
| Occipital isolated atrophy/hypometabolism | No abnormality | 47 (47.5) | 22 (44.0) | - | - |
|  | Any congruence and non-isolated incongruence | 11 (11.1) | 8 (16.0) | 1.55 (0.55-4.40, p=0.407) | 0.05 (0.01-0.46, p=0.009) |
|  | Isolated hypometabolism | 15 (15.2) | 9 (18.0) | 1.28 (0.49-3.38, p=0.616) | 0.18 (0.03-1.05, p=0.057) |
|  | Isolated atrophy | 26 (26.3) | 11 (22.0) | 0.90 (0.38-2.15, p=0.819) | 0.41 (0.11-1.53, p=0.187) |

Isolated atrophy/hypometabolism patterns and progression in MCI/dementia (cut point z=-0.5)

| Variable | Level | Stable | Progressed | OR (univariable) | OR (multivariable) |
| --- | --- | --- | --- | --- | --- |
| Age | Mean (SD) | 71.6 (8.3) | 73.3 (8.9) | 1.02 (0.98-1.07, p=0.284) | 1.03 (0.97-1.09, p=0.343) |
| Sex | Female | 29 (44.6) | 26 (53.1) | - | - |
|  | Male | 36 (55.4) | 23 (46.9) | 0.71 (0.34-1.50, p=0.372) | 0.39 (0.09-1.64, p=0.198) |
| +Atrophy, -Hypometabolism | 0-1 affected lobes | 39 (60.0) | 34 (69.4) | - | - |
|  | 2 or more affected lobes | 26 (40.0) | 15 (30.6) | 0.66 (0.30-1.45, p=0.302) | 0.06 (0.01-0.54, p=0.012) |
| -Atrophy, +Hypometabolism | 0-1 affected lobes | 43 (66.2) | 32 (65.3) | - | - |
|  | 2 or more affected lobes | 22 (33.8) | 17 (34.7) | 1.04 (0.48-2.27, p=0.925) | 1.07 (0.13-8.99, p=0.950) |
| +Atrophy, +Hypometabolism | 0-1 affected lobes | 48 (73.8) | 32 (65.3) | - | - |
|  | 2 or more affected lobes | 17 (26.2) | 17 (34.7) | 1.50 (0.67-3.36, p=0.325) | 0.66 (0.07-5.95, p=0.708) |
| Frontal isolated atrophy/hypometabolism | No abnormality | 30 (46.2) | 18 (36.7) | - | - |
|  | Any congruence and non-isolated incongruence | 9 (13.8) | 13 (26.5) | 2.41 (0.86-6.75, p=0.095) | 0.89 (0.12-6.39, p=0.907) |
|  | Isolated hypometabolism | 13 (20.0) | 9 (18.4) | 1.15 (0.41-3.24, p=0.786) | 0.36 (0.06-2.25, p=0.276) |
|  | Isolated atrophy | 13 (20.0) | 9 (18.4) | 1.15 (0.41-3.24, p=0.786) | 1.24 (0.22-7.13, p=0.806) |
| Temporal isolated atrophy/hypometabolism | No abnormality | 29 (44.6) | 18 (36.7) | - | - |
|  | Any congruence and non-isolated incongruence | 13 (20.0) | 16 (32.7) | 1.98 (0.78-5.07, p=0.153) | 4.75 (0.51-44.61, p=0.173) |
|  | Isolated hypometabolism | 9 (13.8) | 9 (18.4) | 1.61 (0.54-4.82, p=0.393) | 3.57 (0.45-28.05, p=0.226) |
|  | Isolated atrophy | 14 (21.5) | 6 (12.2) | 0.69 (0.22-2.12, p=0.518) | 0.57 (0.09-3.45, p=0.542) |
| Parietal isolated atrophy/hypometabolism | No abnormality | 31 (47.7) | 13 (26.5) | - | - |
|  | Any congruence and non-isolated incongruence | 12 (18.5) | 15 (30.6) | 2.98 (1.10-8.08, p=0.032) | 13.83 (1.48-129.27, p=0.021) |
|  | Isolated hypometabolism | 11 (16.9) | 10 (20.4) | 2.17 (0.74-6.34, p=0.158) | 8.90 (1.03-76.73, p=0.047) |
|  | Isolated atrophy | 11 (16.9) | 11 (22.4) | 2.38 (0.83-6.86, p=0.107) | 33.70 (3.95-287.20, p=0.001) |
| Occipital isolated atrophy/hypometabolism | No abnormality | 25 (38.5) | 22 (44.9) | - | - |
|  | Any congruence and non-isolated incongruence | 9 (13.8) | 8 (16.3) | 1.01 (0.33-3.07, p=0.986) | 0.03 (0.00-0.35, p=0.006) |
|  | Isolated hypometabolism | 15 (23.1) | 9 (18.4) | 0.68 (0.25-1.86, p=0.455) | 0.05 (0.00-0.55, p=0.015) |
|  | Isolated atrophy | 16 (24.6) | 10 (20.4) | 0.71 (0.27-1.88, p=0.492) | 0.39 (0.08-1.94, p=0.249) |

Atrophy/hypometabolism patterns and progression - simple model (varying number of lobes)

| Variable | Level | Stable | Progressed | OR (univariable) | OR (multivariable) |
| --- | --- | --- | --- | --- | --- |
| Age | Mean (SD) | 68.6 (9.7) | 73.6 (9.2) | 1.06 (1.02-1.10, p=0.004) | 1.05 (1.00-1.09, p=0.030) |
| Sex | Female | 50 (50.5) | 27 (54.0) | - | - |
|  | Male | 49 (49.5) | 23 (46.0) | 0.87 (0.44-1.72, p=0.687) | 1.18 (0.48-2.85, p=0.721) |
| +Atrophy, -Hypometabolism | 0 affected lobes | 49 (49.5) | 28 (56.0) | - | - |
|  | 1 or more affected lobes | 50 (50.5) | 22 (44.0) | 0.77 (0.39-1.53, p=0.454) | 0.93 (0.42-2.05, p=0.854) |
| -Atrophy, +Hypometabolism | 0 affected lobes | 55 (55.6) | 23 (46.0) | - | - |
|  | 1 or more affected lobes | 44 (44.4) | 27 (54.0) | 1.47 (0.74-2.91, p=0.271) | 1.23 (0.52-2.92, p=0.643) |
| +Atrophy, +Hypometabolism | 0 affected lobes | 56 (56.6) | 13 (26.0) | - | - |
|  | 1 or more affected lobes | 43 (43.4) | 37 (74.0) | 3.71 (1.76-7.82, p=0.001) | 3.22 (1.41-7.31, p=0.005) |

Atrophy/hypometabolism patterns and progression – simple model (varying number of lobes)

| Variable | Level | Stable | Progressed | OR (univariable) | OR (multivariable) |
| --- | --- | --- | --- | --- | --- |
| Age | Mean (SD) | 68.6 (9.7) | 73.6 (9.2) | 1.06 (1.02-1.10, p=0.004) | 1.05 (1.01-1.09, p=0.025) |
| Sex | Female | 50 (50.5) | 27 (54.0) | - | - |
|  | Male | 49 (49.5) | 23 (46.0) | 0.87 (0.44-1.72, p=0.687) | 1.37 (0.53-3.51, p=0.514) |
| +Atrophy, -Hypometabolism | 0-2 affected lobes | 67 (67.7) | 37 (74.0) | - | - |
|  | 3 or more affected lobes | 32 (32.3) | 13 (26.0) | 0.74 (0.34-1.57, p=0.428) | 1.41 (0.54-3.71, p=0.485) |
| -Atrophy, +Hypometabolism | 0-2 affected lobes | 75 (75.8) | 36 (72.0) | - | - |
|  | 3 or more affected lobes | 24 (24.2) | 14 (28.0) | 1.22 (0.56-2.62, p=0.620) | 1.78 (0.64-4.92, p=0.267) |
| +Atrophy, +Hypometabolism | 0-2 affected lobes | 74 (74.7) | 23 (46.0) | - | - |
|  | 3 or more affected lobes | 25 (25.3) | 27 (54.0) | 3.47 (1.70-7.12, p=0.001) | 3.88 (1.57-9.61, p=0.003) |
