# Supplementary material for: Prognostic value of complementary biomarkers of neurodegeneration in a mixed memory clinic cohort
Source: PeerJ. 2020 Jul 9;8:e9498. doi: 10.7717/peerj.9498 (PMC7354835; doi:10.7717/peerj.9498)
Supplement: Supplemental Information 4 [file peerj-08-9498-s004.docx]

**A**: Atrophy/hypometabolism patterns and progression - simple model with MMSE outcome

| Variable | Level | Stable | Progressed | OR (univariable) | OR (multivariable) |
| --- | --- | --- | --- | --- | --- |
| Age | Mean (SD) | 68.7 (9.8) | 73.2 (9.3) | 1.05 (1.01-1.09, p=0.011) | 1.03 (0.99-1.08, p=0.106) |
| Sex | Female | 45 (45.5) | 29 (61.7) | - | - |
|  | Male | 54 (54.5) | 18 (38.3) | 0.52 (0.25-1.05, p=0.068) | 0.79 (0.32-1.99, p=0.622) |
| +Atrophy, -Hypometabolism | 0-1 affected lobes | 60 (60.6) | 29 (61.7) | - | - |
|  | 2 or more affected lobes | 39 (39.4) | 18 (38.3) | 0.95 (0.47-1.95, p=0.899) | 1.08 (0.47-2.48, p=0.855) |
| -Atrophy, +Hypometabolism | 0-1 affected lobes | 64 (64.6) | 33 (70.2) | - | - |
|  | 2 or more affected lobes | 35 (35.4) | 14 (29.8) | 0.78 (0.37-1.64, p=0.506) | 0.97 (0.37-2.54, p=0.953) |
| +Atrophy, +Hypometabolism | 0-1 affected lobes | 68 (68.7) | 16 (34.0) | - | - |
|  | 2 or more affected lobes | 31 (31.3) | 31 (66.0) | 4.25 (2.03-8.89, p<0.001) | 3.37 (1.49-7.60, p=0.003) |

**B**: Atrophy/hypometabolism patterns and progression - simple model with educational level and disease duration

| Variable | Level | Stable | Progressed | OR (univariable) | OR (multivariable) |
| --- | --- | --- | --- | --- | --- |
| Age | Mean (SD) | 68.6 (9.7) | 73.6 (9.2) | 1.06 (1.02-1.10, p=0.004) | 1.05 (1.00-1.09, p=0.048) |
| Sex | Female | 50 (50.5) | 27 (54.0) | - | - |
|  | Male | 49 (49.5) | 23 (46.0) | 0.87 (0.44-1.72, p=0.687) | 1.74 (0.67-4.53, p=0.259) |
| +Atrophy, -Hypometabolism | 0-1 affected lobes | 58 (58.6) | 32 (64.0) | - | - |
|  | 2 or more affected lobes | 41 (41.4) | 18 (36.0) | 0.80 (0.39-1.61, p=0.524) | 1.17 (0.50-2.76, p=0.715) |
| -Atrophy, +Hypometabolism | 0-1 affected lobes | 67 (67.7) | 32 (64.0) | - | - |
|  | 2 or more affected lobes | 32 (32.3) | 18 (36.0) | 1.18 (0.58-2.41, p=0.654) | 1.06 (0.40-2.81, p=0.907) |
| +Atrophy, +Hypometabolism | 0-1 affected lobes | 69 (69.7) | 17 (34.0) | - | - |
|  | 2 or more affected lobes | 30 (30.3) | 33 (66.0) | 4.46 (2.16-9.22, p<0.001) | 4.98 (2.09-11.86, p<0.001) |
| Disease duration (yrs) | Mean (SD) | 2.6 (3.0) | 2.1 (1.4) | 0.92 (0.79-1.08, p=0.328) | 0.94 (0.80-1.12, p=0.509) |
| Educational level | Low | 24 (24.2) | 13 (26.0) | - | - |
|  | Mid | 30 (30.3) | 19 (38.0) | 1.17 (0.48-2.84, p=0.730) | 2.39 (0.85-6.77, p=0.100) |
|  | High | 45 (45.5) | 18 (36.0) | 0.74 (0.31-1.76, p=0.494) | 1.51 (0.54-4.22, p=0.434) |
