# Supplementary material for: Prognostic value of complementary biomarkers of neurodegeneration in a mixed memory clinic cohort
Source: PeerJ. 2020 Jul 9;8:e9498. doi: 10.7717/peerj.9498 (PMC7354835; doi:10.7717/peerj.9498)
Supplement: Supplemental Information 5 [file peerj-08-9498-s005.docx]

| Variable | Level | Stable | Progressed | Odds ratio (univariable) | Odds ratio (multivariable) |
| --- | --- | --- | --- | --- | --- |
| Age | Mean (SD) | 71.6 (8.3) *^‡^* | 73.3 (8.9) *^§^* | 1.02 (0.98-1.07, p=0.284) | 1.01 (0.97-1.06, p=0.551) |
| Sex | Female | 29 (44.6) | 26 (53.1) | - | - |
|  | Male | 36 (55.4) | 23 (46.9) | 0.71 (0.34-1.50, p=0.372) | 1.15 (0.44-3.02, p=0.775) |
| +Atrophy, -Hypometabolism* | 0-1 affected lobes | 43 (66.2) | 32 (65.3) | - | - |
|  | 2 or more affected lobes | 22 (33.8) | 17 (34.7) | 1.04 (0.48-2.27, p=0.925) | 1.20 (0.49-2.96, p=0.694) |
| -Atrophy, +Hypometabolism | 0-1 affected lobes | 40 (61.5) | 31 (63.3) | - | - |
|  | 2 or more affected lobes | 25 (38.5) | 18 (36.7) | 0.93 (0.43-2.00, p=0.851) | 1.11 (0.43-2.90, p=0.826) |
| +Atrophy, +Hypometabolism | 0-1 affected lobes | 39 (60.0) | 17 (34.7) | - | - |
|  | 2 or more affected lobes | 26 (40.0) | 32 (65.3) | 2.82 (1.31-6.10, p=0.008) | 2.91 (1.22-6.97, p=0.016) |

^*^+ refers to a z-score< 0 in an affected lobe (right and left hemisphere frontal, temporal, parietal and/or occipital) for either hypometabolism ([^18^F]FDG-PET uptake) and/or atrophy (MRI volume). ^‡^n (% of stable) if nothing else stated under level. ^§^n (% of progressed) if nothing else stated under level.
