# Supplementary material for: Prognostic value of complementary biomarkers of neurodegeneration in a mixed memory clinic cohort
Source: PeerJ. 2020 Jul 9;8:e9498. doi: 10.7717/peerj.9498 (PMC7354835; doi:10.7717/peerj.9498)
Supplement: Supplemental Information 6 [file peerj-08-9498-s006.docx]

| Variable | Level | Stable | Progressed | Odds ratio (univariable) | Odds ratio (multivariable) |
| --- | --- | --- | --- | --- | --- |
| Age | Mean (SD) | 71.6 (8.3)^‡^ | 73.3 (8.9)^§^ | 1.02 (0.98-1.07, p=0.284) | 1.01 (0.96-1.07, p=0.709) |
| Sex | Female | 29 (44.6) | 26 (53.1) | - | - |
|  | Male | 36 (55.4) | 23 (46.9) | 0.71 (0.34-1.50, p=0.372) | 1.16 (0.34-3.95, p=0.815) |
| +Atrophy, -Hypometabolism^*^ | 0-1 affected lobes | 43 (66.2) | 32 (65.3) | - | - |
|  | 2 or more affected lobes | 22 (33.8) | 17 (34.7) | 1.04 (0.48-2.27, p=0.925) | 2.36 (0.42-13.11, p=0.326) |
| -Atrophy, +Hypometabolism | 0-1 affected lobes | 40 (61.5) | 31 (63.3) | - | - |
|  | 2 or more affected lobes | 25 (38.5) | 18 (36.7) | 0.93 (0.43-2.00, p=0.851) | 0.76 (0.17-3.44, p=0.723) |
| +Atrophy, +Hypometabolism | 0-1 affected lobes | 39 (60.0) | 17 (34.7) | - | - |
|  | 2 or more affected lobes | 26 (40.0) | 32 (65.3) | 2.82 (1.31-6.10, p=0.008) | 3.52 (0.54-23.14, p=0.190) |
| Frontal isolated atrophy/hypometabolism | No abnormality | 13 (20.0) | 4 (8.2) | - | - |
|  | Any congruence and/or non-isolated atrophy/hypometabolism^†^ | 16 (24.6) | 25 (51.0) | 5.08 (1.41-18.34, p=0.013) | 3.49 (0.42-28.96, p=0.246) |
|  | Isolated hypometabolism | 18 (27.7) | 14 (28.6) | 2.53 (0.67-9.47, p=0.169) | 2.69 (0.55-13.10, p=0.220) |
|  | Isolated atrophy | 18 (27.7) | 6 (12.2) | 1.08 (0.25-4.63, p=0.914) | 0.52 (0.05-5.47, p=0.588) |
| Temporal isolated atrophy/hypometabolism | No abnormality | 13 (20.0) | 6 (12.2) | - | - |
|  | Any congruence and/or non-isolated atrophy/hypometabolism | 25 (38.5) | 29 (59.2) | 2.51 (0.83-7.59, p=0.102) | 1.58 (0.19-13.46, p=0.673) |
|  | Isolated hypometabolism | 16 (24.6) | 8 (16.3) | 1.08 (0.30-3.92, p=0.903) | 1.34 (0.15-11.90, p=0.795) |
|  | Isolated atrophy | 11 (16.9) | 6 (12.2) | 1.18 (0.30-4.73, p=0.813) | 2.45 (0.25-23.46, p=0.438) |
| Parietal isolated atrophy/hypometabolism | No abnormality | 12 (18.5) | 7 (14.3) | - | - |
|  | Any congruence and/or non-isolated atrophy/hypometabolism | 22 (33.8) | 27 (55.1) | 2.10 (0.71-6.25, p=0.181) | 1.18 (0.16-8.70, p=0.869) |
|  | Isolated hypometabolism | 18 (27.7) | 10 (20.4) | 0.95 (0.28-3.20, p=0.937) | 1.44 (0.22-9.42, p=0.704) |
|  | Isolated atrophy | 13 (20.0) | 5 (10.2) | 0.66 (0.16-2.65, p=0.557) | 0.81 (0.08-7.87, p=0.855) |
| Occipital isolated atrophy/hypometabolism | No abnormality | 11 (16.9) | 9 (18.4) | - | - |
|  | Any congruence and/or non-isolated atrophy/hypometabolism | 25 (38.5) | 21 (42.9) | 1.03 (0.36-2.95, p=0.961) | 0.16 (0.02-1.37, p=0.094) |
|  | Isolated hypometabolism | 15 (23.1) | 10 (20.4) | 0.81 (0.25-2.68, p=0.736) | 0.44 (0.06-3.13, p=0.411) |
|  | Isolated atrophy | 14 (21.5) | 9 (18.4) | 0.79 (0.23-2.65, p=0.697) | 0.42 (0.05-3.47, p=0.418) |

^*^+ refers to a z-score< 0 in an affected lobe (right and left hemisphere frontal, temporal, parietal and/or occipital) for either hypometabolism ([^18^F]FDG-PET uptake) and/or atrophy (MRI volume). ^†^Congruence refers to coexisting hypometabolism ([^18^F]FDG-PET z-score<0) and atrophy (MRI z-score<0) in a specific region (left and/or right hemisphere). Incongruence refers to either [^18^F]FDG-PET and MRI z-score<0. Isolation means that the presence of either [^18^F]FDG-PET or MRI z-score<0 in a region (frontal, temporal, parietal or occipital) were without the presence of the other. No abnormality = [^18^F]FDG-PET and MRI z-score>0 for both hemispheres. ^‡^value in parentheses is % of stable if nothing else stated under level. ^‡^n (% of stable) if nothing else stated under level. ^§^n (% of progressed) if nothing else stated under level.
