# Supplementary material for: Prognostic value of complementary biomarkers of neurodegeneration in a mixed memory clinic cohort
Source: PeerJ. 2020 Jul 9;8:e9498. doi: 10.7717/peerj.9498 (PMC7354835; doi:10.7717/peerj.9498)
Supplement: Supplemental Information 7 [file peerj-08-9498-s007.pdf]

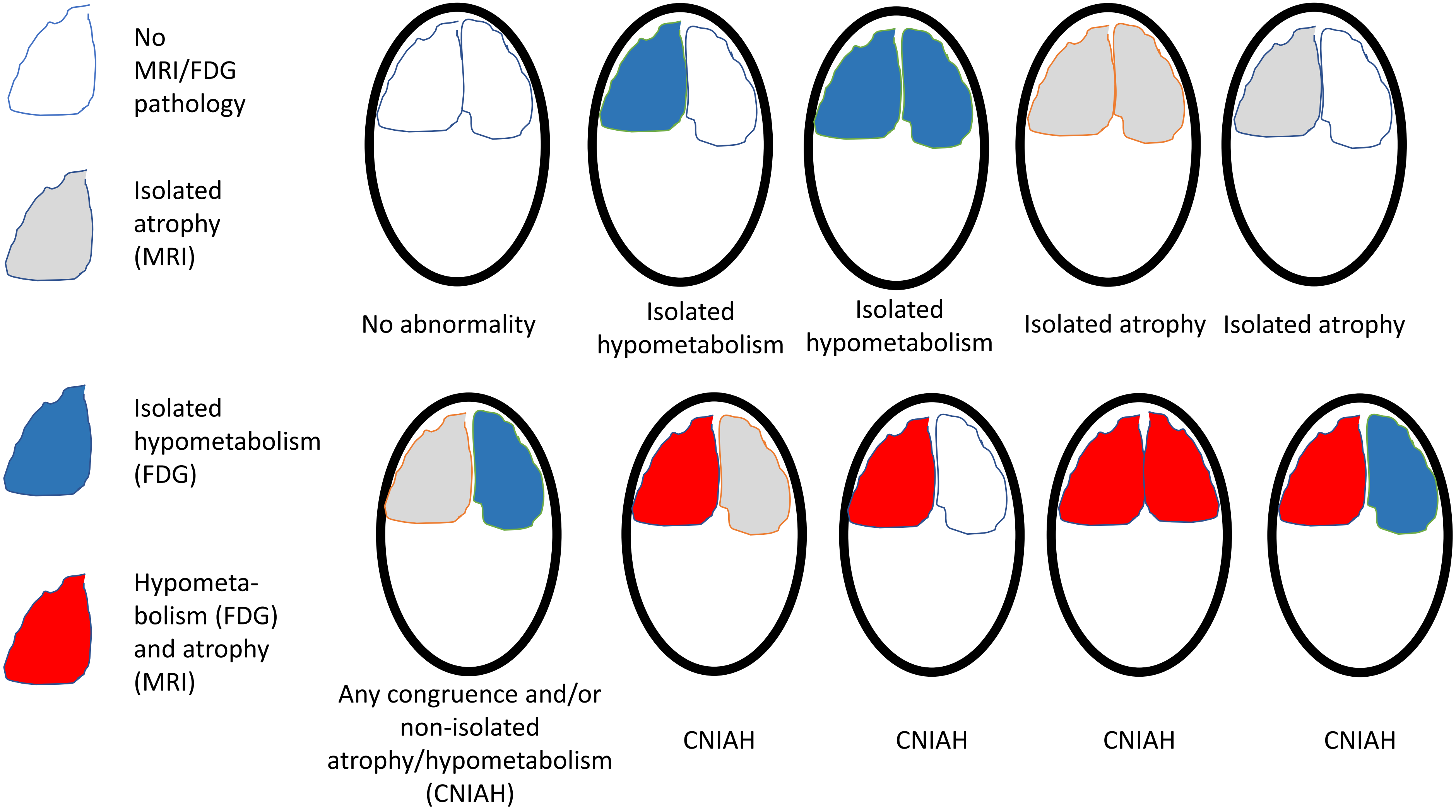
No MRI/FDG pathology
Isolated atrophy (MRI)
Isolated hypometabolism (FDG)
Hypometa-bolism (FDG) and atrophy (MRI)
No abnormality
Isolated hypometabolism
Isolated hypometabolism
Isolated atrophy
Isolated atrophy
Any congruence and/or non-isolated atrophy/hypometabolism (CNIAH)
CNIAH
CNIAH
CNIAH
CNIAH
