# Supplementary figures and images for: Prognostic value of complementary biomarkers of neurodegeneration in a mixed memory clinic cohort
Source: PeerJ. 2020 Jul 9;8:e9498. doi: 10.7717/peerj.9498 (PMC7354835; doi:10.7717/peerj.9498)

Total ventricular volume vs. MMSE difference

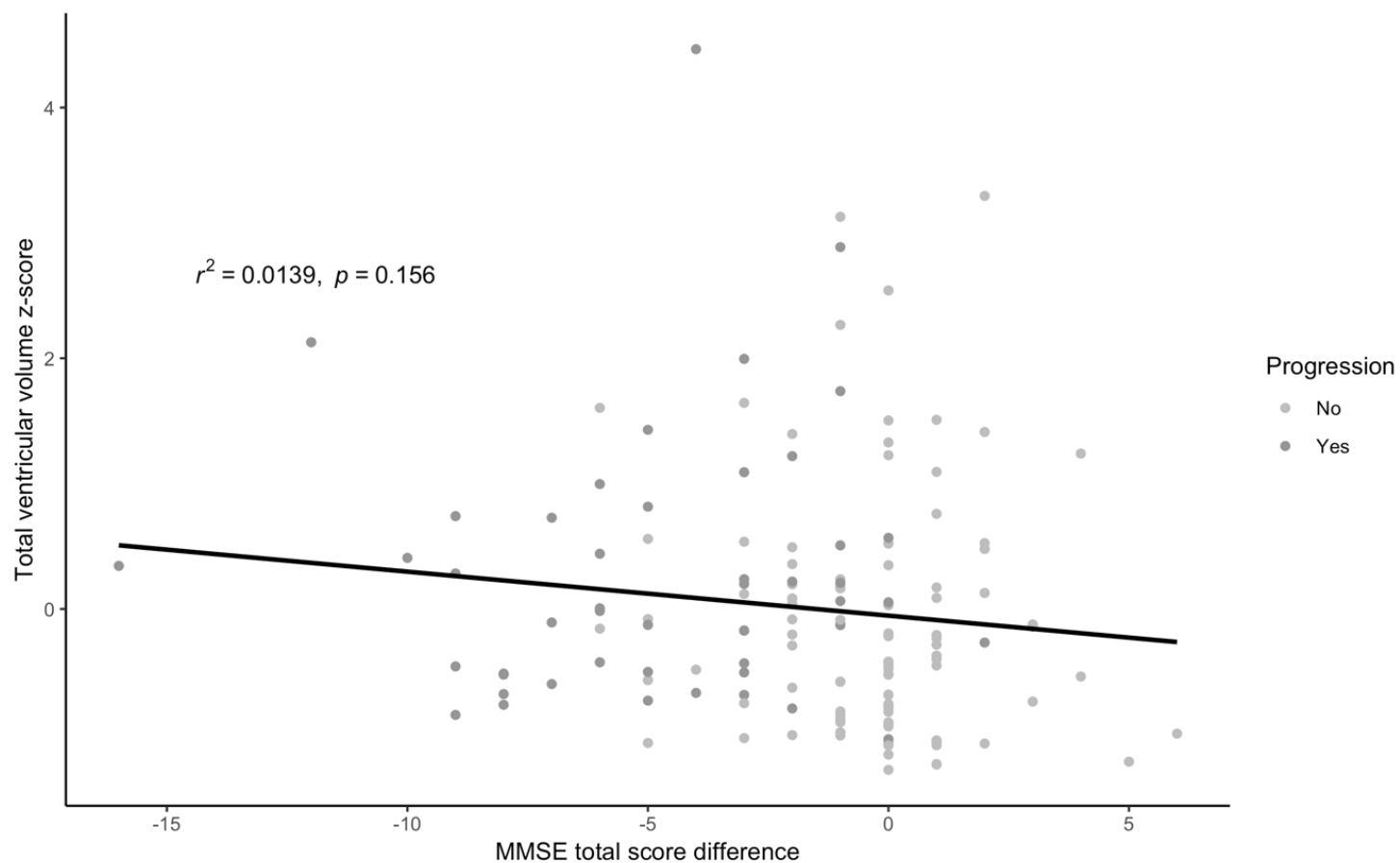

Supplement: Supplemental Information 8 [file peerj-08-9498-s008.pdf]

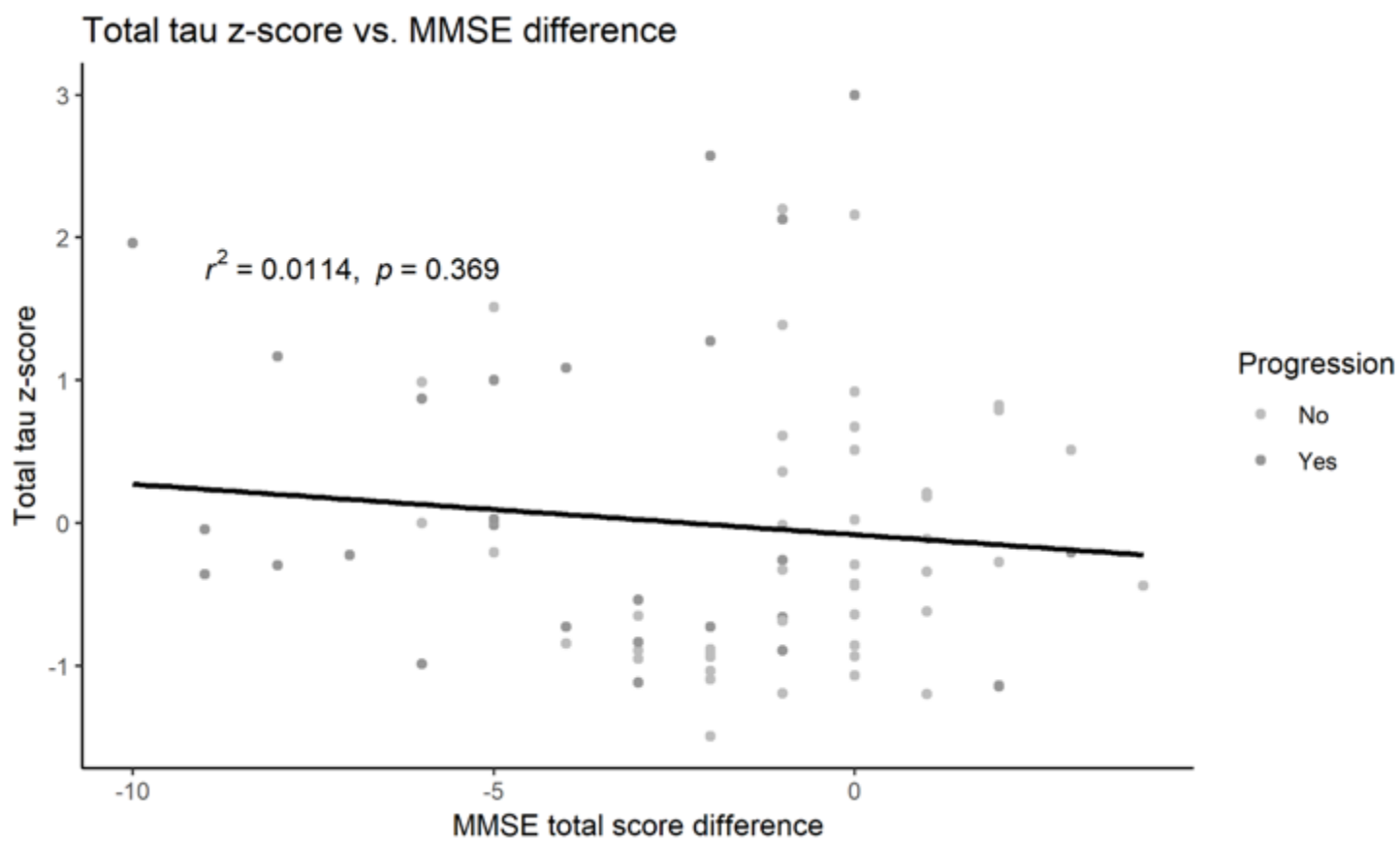

Supplement: Supplemental Information 9 [file peerj-08-9498-s009.pdf]

Grey and white matter total FDG uptake vs. MMSE difference

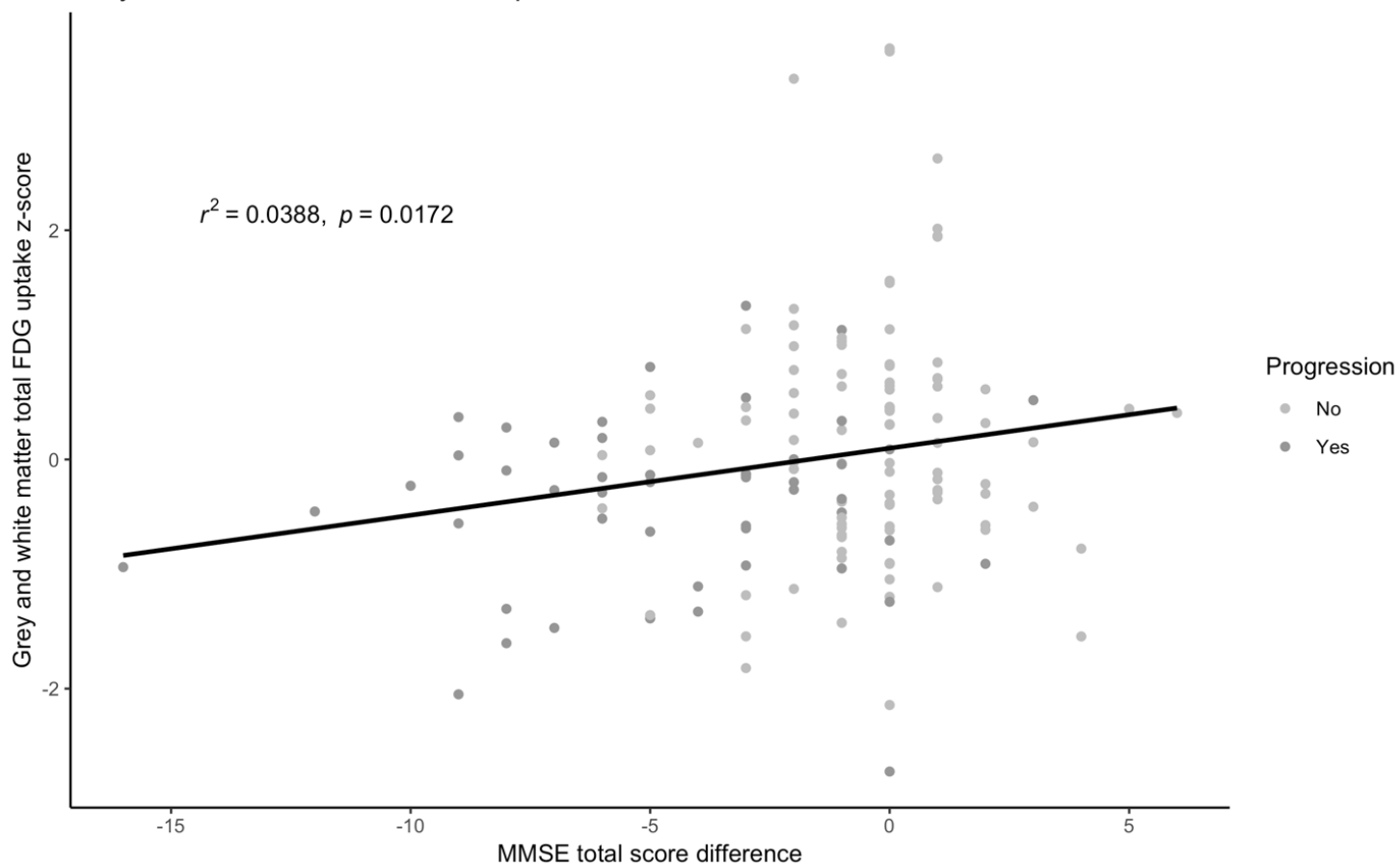

Supplement: Supplemental Information 10 [file peerj-08-9498-s010.pdf]
